# Varicella vaccination in Europe – taking the practical approach

**DOI:** 10.1186/1741-7015-7-26

**Published:** 2009-05-28

**Authors:** Paolo Bonanni, Judith Breuer, Anne Gershon, Michael Gershon, Waleria Hryniewicz, Vana Papaevangelou, Bernard Rentier, Hans Rümke, Catherine Sadzot-Delvaux, Jacques Senterre, Catherine Weil-Olivier, Peter Wutzler

**Affiliations:** 1 Department of Public Health, University of Florence, Florence, Italy; 2 Skin Virus Laboratory, Centre for Cutaneous Research, St Bartholomew's and The Royal London School of Medicine and Dentistry, Queen Mary College, London, UK; 3 Department of Pediatrics, Columbia University College of Physicians and Surgeons, New York, USA; 4 Department of Pathology and Cell Biology, Columbia University College of Physicians and Surgeons, New York, USA; 5 National Medicines Institute, Chelmska Street, 00-725 Warsaw, Poland; 6 Second Department of Pediatrics, University of Athens Medical School, "P & A Kyriakou" Children's Hospital, Athens, Greece; 7 Unit of Fundamental Virology and Immunology, GIGA-Research, B34 University of Liége, 4000 Liège, Belgium; 8 Vaxinostics, University Vaccine Center Rotterdam Nijmegen, Rotterdam, the Netherlands; 9 Department of Pediatrics, University of Liège, Liège, Belgium; 10 University Paris VII, Paris 75005, France; 11 Institute of Virology and Antiviral Therapy, Friedrich-Schiller University, Jena, Germany

## Abstract

Varicella is a common viral disease affecting almost the entire birth cohort. Although usually self-limiting, some cases of varicella can be serious, with 2 to 6% of cases attending a general practice resulting in complications. The hospitalisation rate for varicella in Europe ranges from 1.3 to 4.5 per 100,000 population/year and up to 10.1% of hospitalised patients report permanent or possible permanent sequelae (for example, scarring or ataxia). However, in many countries the epidemiology of varicella remains largely unknown or incomplete.

In countries where routine childhood vaccination against varicella has been implemented, it has had a positive effect on disease prevention and control. Furthermore, mathematical models indicate that this intervention strategy may provide economic benefits for the individual and society. Despite this evidence and recommendations for varicella vaccination by official bodies such as the World Health Organization, and scientific experts in the field, the majority of European countries (with the exception of Germany and Greece) have delayed decisions on implementation of routine childhood varicella vaccination, choosing instead to vaccinate high-risk groups or not to vaccinate at all.

In this paper, members of the Working Against Varicella in Europe group consider the practicalities of introducing routine childhood varicella vaccination in Europe, discussing the benefits and challenges of different vaccination options (vaccination *vs*. no vaccination, routine vaccination of infants *vs*. vaccination of susceptible adolescents or adults, two doses *vs*. one dose of varicella vaccine, monovalent varicella vaccines *vs*. tetravalent measles, mumps, rubella and varicella vaccines, as well as the optimal interval between two doses of measles, mumps, rubella and varicella vaccines).

Assessment of the epidemiology of varicella in Europe and evidence for the effectiveness of varicella vaccination provides support for routine childhood programmes in Europe. Although European countries are faced with challenges or uncertainties that may have delayed implementation of a childhood vaccination programme, many of these concerns remain hypothetical and with new opportunities offered by combined measles, mumps, rubella and varicella vaccines, reassessment may be timely.

## Background

Varicella is a highly contagious disease caused by the varicella zoster virus (VZV), a member of the alpha herpesvirus family. Varicella (or chickenpox) is characterised by a vesicular rash, usually accompanied by fever and malaise [1].

In 1998, the World Health Organization (WHO) recommended that routine childhood varicella vaccination be considered in countries where the disease is a relatively important public health and socioeconomic problem, where the vaccine is affordable, and where high (85 to 90%) and sustained vaccine coverage can be achieved [2]. More than 10 years later, the varicella vaccine is used in childhood immunisation programmes in Australia [3], Canada [4], Germany [5], Greece [6], Qatar [7], Republic of Korea [8], Saudi Arabia [7], Taiwan [9], United States of America (USA) [10], Uruguay [11] and parts of Italy (Sicily) [8] and Spain (Autonomous Community of Madrid) [12].

However, recommendations for varicella vaccination in Europe vary, with the majority of countries not following the WHO recommendations for universal routine vaccination (URV), instead recommending vaccination of susceptible adolescents or high-risk groups. Furthermore, despite the proven health benefits of varicella vaccination [11,13-16], most European countries have delayed the introduction of the vaccine into their immunisation schedules.

This paper seeks to explore the basis of the WHO recommendation in relation to the clinical epidemiology of varicella in Europe, and to understand the reasons for the recommendations not being widely adopted in this region. The practicalities of introducing varicella vaccination into national childhood immunisation calendars in Europe are also evaluated, including discussion on the benefits and challenges of different schedules for monovalent varicella vaccines (*Varilrix*™, GlaxoSmithKline Biologicals, Rixensart, Belgium and *Varivax*™ Sanofi Pasteur MSD, Lyon, France) and for the new combination measles, mumps, rubella, varicella (MMRV) vaccines (*Priorix-Tetra™*, GlaxoSmithKline Biologicals, Rixensart, Belgium and *ProQuad*™, Sanofi Pasteur MSD, Lyon, France).

### Why vaccinate against varicella in Europe?

The clinical epidemiology of varicella in Europe is summarised in Table S1 (Additional file 1) (mortality) [17-31] and Additional file 2 (incidence, hospitalisations and complications) [12,17-27,29,30,32-43]. It is noteworthy that differences in study methodology mean that epidemiological information cannot be easily compared across studies. Furthermore, the majority of studies were conducted in hospitals, with few data available from non-hospitalised patients. In many European countries, such as Belgium and the United Kingdom (UK), the true clinical impact of varicella remains to be determined because it is not a notifiable disease. In other countries, epidemiological data are available, but they may not accurately reflect the true burden of varicella given under-reporting to statutory notification systems [38]. For example, in Italy, the age-standardised incidence of varicella in children 0 to14 years of age collected from a paediatric sentinel network of primary care physicians was 3.8-fold higher than that from statutory notifications (*P *< 0.0001) [38].

### Varicella incidence and seroprevalence

Population surveys described in Additional file 2 reveal that varicella is a common disease in European countries affecting nearly the entire birth cohort. For example, in Germany there was an annual incidence of approximately 760,000 new varicella cases in a birth cohort of around 800,000 prior to widespread vaccination in 1999 [35].

In Europe, the sero-epidemiology of VZV characterises varicella as a disease of childhood, with rapid acquisition of antibodies to VZV during early life [24,44-52]. By adolescence, the majority of individuals are seropositive for VZV antibodies [44,45]. However, there are differences across the European region [44]; seroprevalence appears to be slightly lower among children in southern European countries [51,52] than in northern or central European countries (Table S2 in Additional file 1) [48,53], which may be explained by different climates (Mediterranean *vs*. continental) [54]. Some European countries, such as the UK and Belgium, report a higher seroprevalence among children younger than 4 years of age than other parts of Europe, attributed to the high-level use of day-care nurseries and pre-school facilities [48,55,56].

### Hospitalisations and complications from varicella

While usually self-limiting, a case of varicella can develop potentially serious complications (estimated in approximately 2 to 6% of cases attending a general practice; Additional file 2) [35,57,58]. Common complications in individuals hospitalised with varicella include superinfection of the skin, respiratory complications and neurological manifestations (for example, cerebellitis and encephalitis) [21,30,32,36,41]. Although seldom reported, long-term (including permanent or possible permanent) sequelae, which include severe scarring, ataxia/coordination disorder, epilepsy and cerebral nerve paralysis, occur in 0.4 to 10.1% of patients hospitalised for varicella (Additional file 2) [20,21,36].

In the European studies identified, the incidence of hospitalisation for varicella ranged from 1.3 to 4.5 per 100,000 population/year [24,27-29,43] and 12.9 to 28.0 per 100,000 children ≤ 16 years/year [17,20,21,32,42], with an average duration of hospital stay ranging from 3 to 8 days (Additional file 2) [19,20,22,24,27,30,33-35,42,43]. Most complications and hospitalisations for varicella occurred in children who were immunologically healthy with no underlying medical conditions [20,23].

### Deaths from varicella

Occasionally, complications of varicella can be fatal; mortality rates from identified European studies are shown in Table S1 (Additional file 1) [17-31]. Of 13 varicella-related deaths reporting in children aged 9 months to 9 years, during the 2006 to 2007 varicella season in England, Scotland and Wales, 12 occurred in immunologically healthy children [59]. Eight children died following catastrophic deterioration over less than 24 hours; group A streptococcal sepsis was confirmed in five children and *Staphylococcus aureus *sepsis in two children [59]. Other potentially fatal complications of varicella in children include pneumonia and myocarditis [60].

The risk of death from varicella is much higher (25- to 174-fold) in adults than in children [17,61]. As in children, the majority of adult deaths from varicella occur in previously healthy individuals, although underlying medical conditions, often immune suppression, contribute to a fatal outcome in some cases [17].

### Varicella during pregnancy

Varicella is a serious infection in pregnancy, estimated to affect about 2,000 pregnancies each year in the UK [62]. If left untreated, pregnant women are at greater risk of varicella pneumonia (occurring in about 9% of pregnant women with varicella) [63]. Varicella may also cause complications in the infant caused by intrauterine infection with VZV *via *placental transmission at any stage of pregnancy. Intrauterine infection of the foetus in the early stages of development may result in congenital varicella syndrome (an incidence of about 2% after maternal varicella in the first 20 weeks of gestation [64]), characterised by skin lesions with a dermatomal distribution, neurological defects, ocular disease and skeletal abnormalities [64]. If the mother develops a varicella rash during the period from 4 days before to 2 days after delivery the infant is at risk of neonatal varicella [65]. If left untreated, generalised neonatal varicella can lead to death in about 20% of cases. The serious consequences of varicella during pregnancy and the increased risk of herpes zoster for the child can be minimised by appropriate diagnosis and by using currently available immunoprophylactic and therapeutic interventions [66].

### Herpes zoster

Following primary infection with VZV, the virus enters a period of latency within the dorsal root ganglia of the nervous system. The latent virus can be reactivated with neuronal transfer to the skin manifesting as herpes zoster. The mechanism of VZV reactivation is poorly understood, but several potential risk factors have been identified [67]. The incidence of herpes zoster increases sharply from 50 to 60 years of age, increasing into later life [68]. Although several studies have shown higher rates of reactivation in immunocompromised patients [67], recent reports suggest that herpes zoster predominantly affects immunologically healthy individuals [58,69].

Initially, herpes zoster manifests as a characteristic rash accompanied by acute pain; however, further complications occur in 21 to 48% of patients [58]. The most common and feared complication of herpes zoster is chronic neuropathic pain, known as post-herpetic neuralgia (PHN) [68]. PHN occurs more frequently with advancing age and is estimated to affect half of patients with herpes zoster aged 70 years or older [70]. It can persist for many years and may have a profound negative effect on patient quality of life, substantially interfering with physical, emotional and social functioning, vitality and mental health [68,71,72].

### Current status of varicella vaccination in Europe

The clinical burden of varicella in Europe demonstrates a medical need for prevention strategies against the disease. However, existing recommendations for varicella vaccination in Europe vary widely between countries (Table S3 in Additional file 1) [6,73-75].

Germany has the widest experience with varicella URV in Europe since use of the monovalent vaccine was recommended by the Ständigen Impfkommission of the Robert Koch-Institut in July 2004 [5]. From 2006, the combined MMRV vaccine was included in the German childhood immunisation calendar as a two-dose schedule to be used in place of MMR and varicella vaccines at the physician's discretion [76]. Preliminary reports are now emerging on the impact of varicella vaccination in Germany. Data from a nationwide sentinel surveillance system showed that between April and September 2005, and April and September 2007 the average number of varicella cases reported per physician declined from 17 to 9 cases, corresponding with an increase in vaccine doses administered from 32 to 62 doses per physician [77]. A decline in varicella-related complications was also observed [77]. The reduction in the number of varicella cases and complications was noted in all age groups, but was most pronounced in infants aged 1 to 2 years. These early findings support previous estimates from epidemiological and economic modelling suggesting that varicella URV would be a highly efficient strategy in Germany to reduce the burden of varicella, resulting in benefits for both the individual and society [78,79].

The majority of European countries with a national recommendation for varicella vaccination suggest targeted vaccination in susceptible adolescents or high-risk groups, such as seronegative women of childbearing age, healthcare workers, susceptible individuals with immunosuppressed close contacts, day-care personnel and teachers (Table S3 in Additional file 1). However, this strategy does not have the potential to interrupt viral transmission and, in the past, has been far less effective in achieving high coverage rates when compared with childhood programmes [74].

### How can varicella vaccination be implemented in Europe?

Given that monovalent varicella vaccines have been available in Europe for more than 20 years, there has already been much debate on how best to introduce varicella vaccination. After consideration of the issues surrounding European varicella epidemiology, the Working Against Varicella in Europe group (then known as EuroVar) published a consensus statement in 2004. This statement recommended varicella vaccination for all children between 12 and 18 months of age, and to older, susceptible individuals if high coverage can be rapidly achieved [80]. The health and economic benefits of varicella vaccination have also been recognised by the Society of Independent European Vaccination Experts who have recently recommended that immunisation of susceptible adolescents should be urgently implemented [74]. However, the Society cautions that URV, preferably incorporating a two-dose schedule, will be needed to finally reduce the burden of varicella disease. The dramatic reduction in varicella morbidity and mortality rates observed in the USA following varicella URV has also led some experts to suggest that a universal varicella vaccination policy may be most appropriate for Europe, not just for the medical benefits that it would provide, but also for its social and economic advantages [81].

The long-awaited availability of MMRV vaccines has reinitiated discussions on how varicella vaccination can be implemented. The EuroVar consensus statement recognised that high coverage rates for varicella vaccination are more likely to be achieved if the vaccine was combined with the existing measles-mumps-rubella (MMR) vaccine (that is, as a tetravalent MMRV vaccine) [80]. Indeed, EuroVar stated that the MMRV vaccine will not only enhance the implementation of varicella URV, but represents the way forward, providing a more convenient method of administration of the vaccine to children. Since 2006, two MMRV vaccines have been licensed in Europe – *Priorix-Tetra*™ and *ProQuad*^®^. Both vaccines are immunogenic with similar reactogenicity profiles compared with established MMR and varicella vaccines [82,83].

### Varicella universal routine vaccination *vs*. no vaccination

In countries where varicella URV has been implemented, it has proven a highly effective strategy for reducing the number of varicella cases, hospitalisations, ambulatory visits and deaths [11,84,85]. Using German epidemiological data from an age-structured dynamic infectious disease model (based on a birth cohort of 800,000 individuals), the likely consequences of delaying decisions on varicella vaccination for 1 year are 739,000 cases of varicella, 39,720 complicated cases, 5,740 hospitalisations and 22 deaths [86].

### Routine childhood varicella vaccination *vs*. adolescent/adult varicella vaccination

Vaccination of susceptible adolescents from age 11 to 12 years onwards is an attractive option for healthcare payers. This strategy can prevent the elevated morbidity and mortality of varicella in older age groups, create a cohort of individuals less likely to develop herpes zoster later in life, mitigate the theoretical increase in herpes zoster with varicella URV and minimise the risk of a potential upward age-shift on the peak incidence of the disease (discussed later) [74]. It could also reduce the risk of congenital varicella syndrome and neonatal varicella by protecting women of childbearing age [74]. In some areas of Southern Europe, it has been suggested that vaccination of adolescents could be based on a negative history for varicella in place of serological testing for varicella antigens, thus minimising the cost of this strategy [52]. For example, in Greece, where a high proportion of adolescents (21.5%) are still susceptible to varicella, a self-reported history was reported to have a high negative predictive value (73.5%), except in adolescents with household exposure to VZV [52].

Some caveats exist with an adolescent/adult varicella vaccination strategy. Adolescents, as a group, do not routinely visit a physician and coverage achieved for vaccines recommended in this age group is generally low [87]. Varicella vaccination uptake in adolescents could be improved in some countries by catch-up programmes (that is, opportunistic vaccination). However, this approach may not work well in countries that do not include varicella vaccine in the national immunisation calendar as physicians will not be familiar with the vaccine.

An adolescent vaccination strategy is likely to be beneficial from an individual's perspective, estimated to prevent around 5% of varicella cases, 5% of complications, 8% of hospitalisations and 9% of deaths from varicella (assuming vaccination of 11 to 12 year-olds, with coverage rising from 10% to 30% over the first 5 years of a vaccination programme) [88]. However, from a societal perspective, an adolescent strategy would have minimal effect on transmission of VZV and no benefit in the youngest infants (that is, younger than 1 year of age with a high risk of complications post-varicella) [74]. A universal childhood varicella immunisation programme implemented in such a way that a high coverage rate is achieved [88], could address these issues and would have maximal impact on disease epidemiology, as has been demonstrated from data in the USA and Uruguay [11,85,89,90].

### Monovalent varicella vaccination *vs*. tetravalent MMRV vaccination of infants and children

MMRV vaccines avoid the need for an additional injection when vaccinating against measles, mumps, rubella and varicella, which is beneficial for the majority of European countries that have an already crowded vaccination schedule and where administration of more than two vaccines in a single visit is usually not acceptable to parents or carers. URV with MMRV vaccines is likely to rapidly improve the uptake of varicella vaccination, in line with the coverage expected for MMR vaccines, thus maximising herd immunity and reducing the risk of an upward shift in the peak age of varicella disease. In addition to these benefits, two doses of MMRV vaccine will naturally facilitate a two-dose varicella vaccination schedule. MMRV vaccines are used in several countries such as the USA, Germany and Australia, and data demonstrating their effectiveness are eagerly awaited.

The recommendation by the USA Advisory Committee on Immunization Practices (ACIP) for two doses of varicella-containing vaccine as part of routine childhood immunisation, officially adopted in 2007, stated a preference for MMRV vaccines over the component MMR and varicella vaccines [10]. However, ACIP removed the preference for the combination MMRV vaccine in 2008 [91], in response to new data from surveillance by the Centers for Disease Control (CDC) and Merck for *ProQuad*^® ^in the USA. These data suggested that the risk of febrile seizures in the 5 to 12 days after vaccination with the first dose of MMRV is higher than with separately administered MMR and varicella vaccines [92]. Of note, febrile seizures are typically of short duration (< 15 minutes) and resolve without sequelae. Furthermore, the USA Food and Drug Administration continue to be satisfied with the safety and efficacy of MMRV vaccine, having not made any changes to its indications for use and stating that the benefits of using the vaccine continue to outweigh the risks [93]. The situation with regard to MMRV vaccination and febrile seizures continues to be monitored closely. Data are not yet available for GlaxoSmithKline's MMRV vaccine and it is currently unclear what these data may show; the two vaccines differ in terms of measles and varicella dose.

A third option for introduction of varicella vaccination that may be considered by policy makers is to administer the first doses of MMR and varicella vaccines separately, followed by a second dose of combined MMRV vaccine at least 4 weeks later [94,95]. This strategy may mitigate any perceived increase in reactogenicity following the first dose of MMRV vaccine.

### Two doses of varicella vaccine *vs*. one dose for universal routine vaccination

Two doses of varicella vaccine as part of the USA childhood immunisation programme have been officially recommended by the ACIP since 2007 [10]. Regulatory approval of MMRV vaccines by the European Medicines Agency also recognises that a second dose of a varicella-containing vaccine ensures maximal protection against the disease [96]. In Europe, two doses of varicella vaccine are recommended in Greece (Table S3 in Additional file 1) and by the Spanish Association of Pediatrics (although this has yet to be officially adopted by the national authorities [97]), and in Germany, MMRV vaccine can be used, resulting in two doses of varicella being given.

Countries considering varicella URV (with monovalent or combined vaccines) have the opportunity to evaluate the merits of a one- or two-dose schedule. In the USA, after a rapid decline in varicella incidence following the introduction of a one-dose vaccination strategy from 1995, the number of cases at CDC surveillance sites reached a plateau in 2002. This occurred despite continued high rates of vaccination coverage [98]. Furthermore, outbreaks of breakthrough varicella (that is, a case of varicella occurring 42 days or more after vaccination following exposure to wild-type virus) continued to occur among immunised children in day-care centres and schools (see recent review by Seward *et al*.) [99]. Most studies in this review showed a varicella vaccine effectiveness of 80 to 89% after one dose [99], thus leaving 10 to 20% of vaccinees who either did not respond to vaccination (primary immune failure) or who experienced waning immunity over time (secondary immune failure) [100]. Two doses of varicella vaccine have been associated with higher vaccine efficacy in clinical studies and the projected risk of breakthrough disease over 10 years is three times lower than among individuals who received one dose [101]. Humoral and cell-mediated responses were also greater after two doses of varicella vaccine, suggesting improved vaccine efficacy compared with one dose [102,103].

### The interval between two doses of varicella vaccine (monovalent or MMRV)

Depending on the product used, the first dose of monovalent varicella vaccine or MMRV vaccine can be administered from the age of 9 or 12 months. The second dose is then administered according to an 'accelerated', 'standard' or 'longer' schedule in accordance with local recommendations. The schedules currently used for MMR vaccines are shown in Table S3 (Additional file 1) [104]. Each strategy is associated with specific benefits and challenges, which are discussed below. For the MMRV vaccine, only the varicella component is discussed – there may be different benefits and challenges for the measles, mumps and rubella components.

### Accelerated schedule: dose 1 at 11 to 23 months and dose 2 at 12 to 24 months

An accelerated schedule means that the two vaccine doses are administered close together (at least 1 month apart; during second year of life for MMR(V) vaccines). This schedule is used for MMR vaccines in Europe in the Czech Republic, Austria, France, Germany and Switzerland [104], and for MMRV vaccines in Germany (Table S3 in Additional file 1) [76]. In the USA, although the recommended timing of the second dose is 4 to 6 years of age, this remains flexible, permitting an accelerated schedule [10], and it is also allowed by the WHO for measles-containing vaccines depending on the local programmatic and epidemiological situation [105].

Administering two doses of varicella-containing vaccine with a short interval provides the opportunity to enhance compliance, and hence vaccine coverage because 2-year-old or younger children are more accessible to healthcare professionals than older age groups. After one dose of vaccine, the risk of breakthrough varicella increases with time. Administering MMRV vaccines according to an accelerated schedule therefore allows high levels of immunity to vaccine antigens to be established early in life, ensuring that, if primary vaccine failure occurs, a child does not remain unprotected for 2 to 12 years (standard and longer schedules).

Clinical studies of varicella and MMRV vaccines show robust and persistent immune responses when two doses are administered according to an accelerated schedule [82,83,101,106]. In one study, in which children were randomised to receive either two doses of MMRV vaccine (*Priorix*-*Tetra*™; MMRV group) or one dose of MMR vaccine (*Priorix*™) concomitantly with varicella vaccine (*Varilrix*™) followed by another MMR vaccination 6 to 8 weeks apart (MMR+V group) [82], seropositivity rates for varicella after 3 years were 99.4% (MMRV group, *N *= 225) and 96.8% (MMR+V group, *N *= 79) [106]. Over the 3-year follow-up period (total vaccinated cohort *N *= 494), two mild varicella breakthrough cases were reported in the MMRV group and four mild and one moderate case in the MMR+V group [106]. No studies evaluating the immune response of two doses of Merck's MMRV vaccine administered according to an accelerated schedule could be identified.

### Standard schedule: dose 1 at 12 to 24 months and dose 2 at 3 to 7 years

A standard schedule with an interval of 1 to 6 years is used for administration of MMR vaccines in the majority of countries in Europe (Table S3 in Additional file 1). In some countries, such as Finland, excellent coverage has been achieved with both vaccine doses, which would be beneficial for varicella vaccination should MMR vaccines be replaced with MMRV vaccines [107]. As for the accelerated schedule, a robust immune response was measured to the second dose of MMRV vaccine given to children at 5 to 6 years [108].

A potential drawback of the standard schedule is the risk of breakthrough disease during the longer interval between vaccine doses. One dose of varicella vaccine was shown to be 80 to 85% effective in a number of field studies, which is not sufficient to prevent outbreaks and interrupt viral transmission [99]. Thus, in the intervening 1 to 6-year period between the two doses, breakthrough cases of varicella can be expected, which could result in outbreaks in close communities such as day-care centres and schools, and cases in susceptible adolescents and adults. However, given the potential scheduling challenges with administering the vaccine according to an accelerated schedule (for example, crowded vaccination calendars during the first 2 years of life), the standard schedule offers an effective alternative.

### Longer schedule: dose 1 at 12 to 18 months and dose 2 at 8 to 13 years

Several countries administer MMR vaccines according to a longer schedule, which means that the two doses are given 7 to 12 years apart (Table S3 in Additional file 1). Administering the second dose of varicella-containing vaccine with a long interval is likely to reduce the risk of waning immunity to vaccine antigens resulting in infection into adolescence and adulthood, when the disease can be more severe [100]. However, as was seen from experiences in the USA, the risk of breakthrough disease between vaccine doses may prevent elimination of varicella. Despite this potential drawback, countries may be hesitant about changing an established longer schedule for fear of a negative impact on vaccine uptake.

### Why has varicella vaccination been delayed in Europe?

There are positive effects of universal childhood varicella vaccination on disease incidence, and the rate of complications and hospitalisations [84]. Given these benefits, it is curious why more countries in Europe have not introduced the varicella vaccine into national immunisation schedules. The possible reasons for this are considered in brief below.

### Lack of recognition of varicella as a serious disease

As noted previously, appreciation of the true burden of varicella can be hampered because varicella is not a notifiable disease in many countries, and there appears to be a degree of underreporting to mandatory notification systems. A lack of awareness about the potential complications of varicella means that the disease continues to be perceived as benign by many physicians and parents.

The available reports of the varicella burden in European countries indicate that children can experience complications of this disease, which may require hospitalisation, and occasionally can be fatal. Communication of varicella outbreaks, such as the large outbreak in the UK in April 2007, may be beneficial in raising awareness of the disease among parents, physicians and public health officials.

### Perception of age shift with varicella vaccines

A concern exists that widespread childhood varicella vaccination may produce an upward shift in the peak age of the disease to older individuals, for whom varicella may be more severe. In the USA there has, as anticipated, been a rise in the age of peak incidence of the disease following the widespread introduction of varicella vaccination with a one-dose schedule. Before 1995, around 73% of varicella cases occurred in children aged 6 years or younger, with a peak disease frequency between 3 and 6 years [100]. In 2004, on the other hand, children aged 6 years and younger accounted for only 30% of varicella cases [100]; disease frequency peaked in vaccinated children (one dose) between the ages of 6 and 9 years and in unvaccinated children between the age of 9 and 12 years [100]. However, the absolute number of cases in older children remained similar to that reported in the pre-vaccination era. Conversely, since URV implementation there has been a decline in the age-specific hospitalisation or varicella incidence rates in all age groups (including infants not eligible for vaccination and adults) [11,90,109]. This observation is most likely explained by herd immunity, where high coverage rates interrupt viral transmission, indirectly protecting those who are not vaccinated.

### Perception of an increase in herpes zoster

A number of sources indicate that boosting of immunity to VZV can reduce the incidence of herpes zoster [67,110]. A case-control study suggested that exogenous exposure to VZV via contact with children protects individuals with latent VZV infection against herpes zoster by boosting immunity [111]. Statistically significant protection (odds ratio 0.29 (95% confidence interval: 0.10 to 0.84)) was achieved for those adults with five or more contacts with VZV during the past 10 years [111]. This type of data led to formulation of the hypothesis that a reduction of childhood varicella by vaccination might lead to increased incidence of herpes zoster in adults [110]. Using mathematical modelling, Brisson *et al *proposed that routine varicella vaccination of children will produce a substantial increase in the rates of herpes zoster over the first 30 to 50 years after vaccination is introduced [110]. In the longer term, under the assumptions of the model, a vaccination programme that eliminates varicella will also eliminate herpes zoster [110]. To date, the majority of studies conducted in countries where the incidence of varicella has been reduced by vaccination have not shown an accompanying increase in herpes zoster [84,109,112,113].

Evidence from different sources suggests that varicella vaccination may be associated with a reduced incidence, and hence lower risk, of herpes zoster in vaccinees [113-116]. In a 10 to 26-year follow-up of individuals vaccinated as healthy young adults, the herpes zoster incidence was 0.91/1,000 person-years, lower than the rates quoted by the authors for unvaccinated individuals (2.15 to 4.05 cases/1,000 person-years) [113]. Furthermore, children with leukaemia (who have a naturally increased risk of herpes zoster), who were vaccinated with one dose of varicella vaccine, had a lower incidence of herpes zoster than children with leukaemia who contracted VZV naturally (crude incidence rates of 8.0 *vs*. 24.6/1,000 person-years, respectively; *P *= 0.01) [114].

Available data support the hypothesis that vaccination may reduce viral latency and thus the incidence of herpes zoster by minimising infection of the skin (that is, the development of varicella lesions) [115], which is thought to be directly related to the establishment of VZV latency [114,115].

In summary, the childhood vaccination programme should reduce the risk of herpes zoster in vaccinees in the longer term. Furthermore, if needed, appropriate action can be taken (for example, implementation of a herpes zoster vaccine in adults).

### Achieving high coverage rates

If varicella vaccination is to be implemented using MMRV vaccines, the existing coverage for MMR vaccines may need to be considered. The WHO recommendations state that coverage rates for varicella vaccination should exceed 85% in order to avoid an upward age-shift in the peak incidence of varicella disease. In European Union countries in 2006, coverage for measles-containing vaccine ranged from 82% to 98% for the first dose, with 18 of 20 countries with data available reporting coverage rates to the WHO exceeding 85% [117]. For the second dose, vaccine coverage ranged from 60 to 98%, with 11 of 14 countries reporting coverage rates to the WHO exceeding 85% [117]. Therefore, the majority of European countries with data available reach the stated threshold of 85% coverage with MMR vaccines, and thus could be considered suitable for implementation of MMRV vaccines.

### Cost (country-specific)

The cost of vaccination is relevant for public health officials and individuals purchasing vaccines on the private market, and is country-specific. European countries apply different models for production of guidelines, and for linking these to funding, but cost-effectiveness is becoming a standard requirement. In some countries, such as the UK and France, only costs from a healthcare perspective are taken into account when considering the cost-effectiveness of vaccination – all societal costs (for example, work-time lost) are excluded from the analysis.

In Germany, findings from a mathematical model suggest that varicella URV with a one-dose schedule will provide essential clinical benefits for individuals, and that significant economic benefits can also be expected [35]. Furthermore, an updated version of this model has assessed the direct and indirect cost-savings associated with two doses of varicella vaccine and MMRV vaccination compared with an adolescent immunisation strategy with one dose of varicella vaccine [118]. The model demonstrated that two doses of MMRV vaccine are cost-saving from a societal and a health system perspective (benefit-cost-ratios [BCR] of 2.56 and 1.08, respectively) [118]. Using the same model, varicella URV with a two-dose schedule was predicted to be highly effective for reducing the burden of varicella disease in Italy, with significant net savings from the societal perspective, but was not cost-saving from the National Health Service perspective in the majority of situations analysed [119].

The latest study results from the USA demonstrated that, compared with no intervention, the two-dose regimen is cost-saving from a societal perspective (BCR = 2.73) (assuming that 30% of children would receive MMRV vaccine as a first dose and 100% would receive MMRV as a second dose, and using 2006 prices) [120]. Furthermore, the economic benefit of the two-dose MMRV regimen compares very favourably with other medical interventions. However, the authors note that compared with one-dose URV, the two-dose MMRV regimen did not produce additional savings from a societal perspective (BCR = 0.56). Notably, however, additional factors such as the cost associated with outbreaks following one-dose URV were not included in the model.

A favourable BCR does not necessarily result in public funding of a vaccine. New vaccines entering the market compete for a share of a limited healthcare budget; in recent years, state-of-the-art vaccines against *Streptococcus pneumoniae*, human papilloma virus and rotavirus have commanded a great deal of public attention and, in some countries, have been prioritised over varicella vaccination.

## Summary

Despite European recommendations for varicella vaccination, VZV continues to cause a high number of varicella cases, potentially requiring medical visits or hospitalisations and occasionally leading to long-term sequelae or even death. The majority of varicella complications occur in healthy children, meaning that it is not possible to predict who will be affected.

With the exception of Germany and Greece, most European countries have delayed the introduction of varicella vaccination into the national immunisation schedule. However, with many concerns about the vaccine remaining hypothetical and the new opportunities offered by MMRV vaccines, reassessment may be timely.

Accumulating evidence from countries that have implemented universal varicella vaccination of infants demonstrates a dramatic reduction in the burden of varicella, thus providing the strongest support for widespread implementation of the WHO recommendation for varicella vaccination in European countries.

## Competing interests

All authors are members of the Working Against Varicella in Europe (WAVE) group. Meetings of this group and the development of this manuscript were supported by an unrestricted educational grant from GlaxoSmithKline Biologicals. PB has participated in the activities of expert groups and boards on vaccines and vaccination policies sponsored by different pharmaceutical companies. JB receives research funding and sponsorship for attending advisory board meetings from GlaxoSmithKline, Merck and Sanofi Pasteur MSD. AG consults and lectures on varicella and zoster vaccines for GlaxoSmithKline and Merck, and also receives research funding from GlaxoSmithKline and Merck. HR/Vaxinostics has performed clinical studies with MMR-varicella vaccines for GSK and Sanofi Pasteur MSD. CWO has participated as an expert in different temporary groups for vaccine manufacturers. PW has been a consultant to GlaxoSmithKline and Sanofi Pasteur MSD, has served on advisory boards for vaccine manufactures and holds a number of shares in GlaxoSmithKline. The following authors declare no other competing interests: MG, WH, VP, BR, CSD, and JS.

## Authors' contributions

All authors contributed to the manuscript by discussing the challenges facing European countries when deciding whether to introduce varicella vaccination, as well as the advantages and disadvantages of different vaccination schedules for monovalent and combined MMRV vaccines at three meetings of the WAVE (Working Against Varicella in Europe) group held in Belgium, Ireland and the Netherlands in December 2007, June 2008 and December 2008, respectively. The WAVE group comprises experts with considerable experience in all aspects of vaccinology, particularly varicella, from 10 European countries (Belgium, Finland, France, Germany, Greece, Italy, Poland, Spain, Netherlands and the UK) and from the USA.

Following the first meeting, a number of resources were searched for articles of relevance to obtain information on the epidemiology of varicella in Europe, including PubMed (using the terms 'MMR', 'MMRV', 'varicella' and 'chickenpox'), respected websites (for example, WHO and CDC) and abstracts from paediatric congresses (for example, European Society for Paediatric Infectious Diseases, International Congress on Infectious Diseases, International Disease Society of America and the World Society for Paediatric Infectious Diseases). Additional information sources were identified from the bibliographies of these references or from the authors' own libraries (national data) and expertise.

The first draft manuscript was prepared by a scientific writer (Dr Alison Lovibond), with contribution from all authors via electronic discussions and face-to-face meetings. The final manuscript was read and approved by all authors.

## Pre-publication history

The pre-publication history for this paper can be accessed here:



## Supplementary Material

Additional file 1 **Tables S1–S3**. Table S1 – Summary of varicella-related mortality data from European countries. Table S2 – Seroprevalence of varicella among children in Europe. Table S3 – Recommendations for varicella and measles-mumps-rubella (MMR) vaccination in European countries. Click here for file

Additional file 2 **Supplementary appendix**. Summary of varicella epidemiology in European countries. Click here for file

## References

[B1] Heininger U, Seward JF (2006). Varicella. Lancet.

[B2] World Health Organization (1998). The WHO position paper on varicella vaccines. Wkly Epidemiol Rec.

[B3] Macartney KK, Burgess MA (2008). Varicella vaccination in Australia and New Zealand. J Infect Dis.

[B4] National Advisory Committee on Immunization (2004). National Advisory Committee on Immunization (NACI) update on varicella. Can Commun Dis Rep.

[B5] Robert Koch Institut (2006). Empfehlungen der Ständigen Impfkommission (STIKO) am Robert Koch-Institut/Stand: Juli 2006. Epidemiol Bulletin.

[B6] Childhood immunisation calendar, Greece. http://www.mohaw.gr/gr/thefuture/anakoinoseis/Timetable%202008.xls.

[B7] WHO vaccine preventable disease monitoring system: Immunization schedules by antigen, selection centre. http://www.who.int/immunization_monitoring/en/globalsummary/scheduleselect.cfm.

[B8] Sadzot-Delvaux C, Rentier B, Wutzler P, Asano Y, Suga S, Yoshikawa T, Plotkin S (2008). Varicella vaccination in Japan, South Korea, and Europe. J Infect Dis.

[B9] Liao SL, Huang T, Huang YC, Jiang DD (2007). Survey of the status of self-paid varicella vaccination among children one to six years of age in Taiwan. J Microbiol Immunol Infect.

[B10] Marin M, Guris D, Chaves SS, Schmid S, Seward JF (2007). Prevention of varicella: recommendations of the Advisory Committee on Immunization Practices (ACIP). MMWR.

[B11] Quian J, Ruttimann R, Romero C, Dall'orso P, Cerisola A, Breuer T, Greenberg M, Verstraeten T (2008). Impact of universal varicella vaccination of one year-olds in Uruguay: 1997–2005. Arch Dis Child.

[B12] Perez-Farinos N, Ordobas M, Garcia-Fernandez C, Garcia-Comas L, Canellas S, Rodero I, Gutierrez-Rodriguez A, Garcia-Gutierrez J, Ramirez R (2007). Varicella and herpes zoster in Madrid, based on the Sentinel General Practitioner Network: 1997–2004. BMC Infect Dis.

[B13] Centers for Disease Control and Prevention (1996). Prevention of varicella: Recommendations of the Advisory Committee on Immunization Practices (ACIP). Centers for Disease Control and Prevention. MMWR.

[B14] Reynolds MA, Watson BM, Plott-Adams KK, Jumaan AO, Galil K, Maupin TJ, Zhang JX, Seward JF (2008). Epidemiology of varicella hospitalizations in the United States, 1995–2005. J Infect Dis.

[B15] Centers for Disease Control and Prevention (2005). Varicella-related deaths – United States, January 2003–June 2004. MMWR.

[B16] Atkinson WHJ, McIntyre L, Wolfe S, Centers for Disease Control and Prevention (2006). Varicella. Epidemiology and Prevention of Vaccine-Preventable Diseases.

[B17] Boelle PY, Hanslik T (2002). Varicella in non-immune persons: incidence, hospitalization and mortality rates. Epidemiol Infect.

[B18] Bonmarin B, Ndiaye B, Seringe E, Levy-Bruh D (2005). Épidémiologie de la varicelle en France. Bull Epidemiol Hebd.

[B19] Mallet E, Maitre M, Delalande-Dutilleul L, Marguet C, Mouterde O (2004). Evaluation of varicella complications through a retrospective hospital survey in a paediatric center over 16 years in France. Arch Pediatr.

[B20] Liese JG, Grote V, Rosenfeld E, Fischer R, Belohradsky BH, v Kries R (2008). The burden of varicella complications before the introduction of routine varicella vaccination in Germany. Pediatr Infect Dis J.

[B21] Theodoridou M, Laina I, Hadjichristodoulou C, Syriopoulou V (2006). Varicella-related complications and hospitalisations in a tertiary pediatric medical center before vaccine introduction. Eur J Pediatr.

[B22] Cameron JC, Allan G, Johnston F, Finn A, Heath PT, Booy R (2007). Severe complications of chickenpox in hospitalised children in the UK and Ireland. Arch Dis Child.

[B23] Marchetto S, de Benedictis FM, de Martino M, Versace A, Chiappini E, Bertaine C, Osimani P, Cordiali R, Gabiano C, Galli L (2007). Epidemiology of hospital admissions for chickenpox in children: an Italian multicentre study in the pre-vaccine era. Acta Paediatr.

[B24] de Melker H, Berbers G, Hahne S, Rumke H, Hof S van den, de Wit A, Boot H (2006). The epidemiology of varicella and herpes zoster in the Netherlands: implications for varicella zoster virus vaccination. Vaccine.

[B25] Boot H, Zanden B van der, van Lier A, Maas N van der, De Melker H, de Melker H, Kramer M (2008). Varicella zoster virus (VZV) infection. The National Immunisation Programme in the Netherlands: developments in 2007.

[B26] Socan M, Blasko M (2007). Surveillance of varicella and herpes zoster in Slovenia, 1996 – 2005. Euro Surveill.

[B27] Gil A, Oyaguez I, Carrasco P, Gonzalez A (2002). Epidemiology of primary varicella hospitalizations in Spain. Vaccine.

[B28] Gil A, Gonzalez A, Oyaguez I, Martin MS, Carrasco P (2004). The burden of severe varicella in Spain, 1995–2000 period. Eur J Epidemiol.

[B29] Gil A, San-Martin M, Carrasco P, Gonzalez A (2004). Epidemiology of severe varicella-zoster virus infection in Spain. Vaccine.

[B30] Bonhoeffer J, Baer G, Muehleisen B, Aebi C, Nadal D, Schaad UB, Heininger U (2005). Prospective surveillance of hospitalisations associated with varicella-zoster virus infections in children and adolescents. Eur J Pediatr.

[B31] Rawson H, Crampin A, Noah N (2001). Deaths from chickenpox in England and Wales 1995–7: analysis of routine mortality data. BMJ.

[B32] Dubos F, Grandbastien B, Hue V, Martinot A (2007). Epidemiology of hospital admissions for paediatric varicella infections: a one-year prospective survey in the pre-vaccine era. Epidemiol Infect.

[B33] Emery C, Lancon F, Fagnani F, Pechevis M, Durand I, Floret D (2006). ENVOL study on the medical management of varicella and its complications in French ambulatory care. Med Mal Infect.

[B34] Grimprel E, Levy C, de La Rocque F, Cohen R, Soubeyrand B, Caulin E, Derrough T, Lecuyer A, d'Athis P, Gaudelus J (2007). Paediatric varicella hospitalisations in France: a nationwide survey. Clin Microbiol Infect.

[B35] Wagenpfeil S, Neiss A, Banz K, Wutzler P (2004). Empirical data on the varicella situation in Germany for vaccination decisions. Clin Microbiol Infect.

[B36] Ziebold C, von Kries R, Lang R, Weigl J, Schmitt HJ (2001). Severe complications of varicella in previously healthy children in Germany: a 1-year survey. Pediatrics.

[B37] Katsafadou A, Ferentinos G, Constantopoulos A, Papaevangelou V (2008). The epidemiology of varicella in school-aged Greek children before the implementation of universal vaccination. Eur J Clin Microbiol Infect Dis.

[B38] Ciofi Degli Atti ML, Salmaso S, Bella A, Arigliani R, Gangemi M, Chiamenti G, Brusoni G, Tozzi AE (2002). Pediatric sentinel surveillance of vaccine-preventable diseases in Italy. Pediatr Infect Dis J.

[B39] Reports on cases of infectious diseases and poisonings in Poland – 2006. http://www.pzh.gov.pl/oldpage/epimeld/2006/index_ma.html.

[B40] Arama V, Rafila A, Streinu-Cercel A, Pistol A, Bacruban R, Sandu R, Pitigoi D, Negoita A (2005). Varicella in Romania: epidemiological trends, 1986–2004. Euro Surveill.

[B41] Diez-Domingo J, Aristegui J, Calbo F, Gonzalez-Hachero J, Moraga F, Pena Guitian J, Ruiz Contreras J, Torrellas A (2003). Epidemiology and economic impact of varicella in immunocompetent children in Spain. A nation-wide study. Vaccine.

[B42] Perez-Yarza EG, Arranz L, Alustiza J, Azkunaga B, Uriz J, Sarasua A, Mendiburu I, Emparanza JI (2003). Hospital admissions for varicella complications in children aged less than 15 years old. An Pediatr (Barc).

[B43] Brisson M, Edmunds WJ (2003). Epidemiology of varicella-zoster virus in England and Wales. J Med Virol.

[B44] Nardone A, de Ory F, Carton M, Cohen D, van Damme P, Davidkin I, Rota MC, de Melker H, Mossong J, Slacikova M, Tischer A, Andrews N, Berbers G, Gabutti G, Gay N, Jones L, Jokinen S, Kafatos G, de Aragon MV, Schneider F, Smetana Z, Vargova B, Vranckx R, Miller E (2007). The comparative sero-epidemiology of varicella zoster virus in 11 countries in the European region. Vaccine.

[B45] Aebi C, Fischer K, Gorgievski M, Matter L, Muhlemann K (2001). Age-specific seroprevalence to varicella-zoster virus: study in Swiss children and analysis of European data. Vaccine.

[B46] Mossong J, Putz L, Schneider F (2004). Seroprevalence and force of infection of varicella-zoster virus in Luxembourg. Epidemiol Infect.

[B47] Salleras L, Dominguez A, Vidal J, Plans P, Salleras M, Taberner JL (2000). Seroepidemiology of varicella-zoster virus infection in Catalonia (Spain). Rationale for universal vaccination programmes. Vaccine.

[B48] Thiry N, Beutels P, Shkedy Z, Vranckx R, Vandermeulen C, Wielen MV, Damme PV (2002). The seroepidemiology of primary varicella-zoster virus infection in Flanders (Belgium). Eur J Pediatr.

[B49] Wutzler P, Farber I, Wagenpfeil S, Bisanz H, Tischer A (2001). Seroprevalence of varicella-zoster virus in the German population. Vaccine.

[B50] Alp H, Altinkaynak S, Ertekin V, Kilicaslan B, Giiraksin A (2005). Seroepidemiology of varicella-zoster virus infection in a cosmopolitan city (Erzurum) in the eastern Turkey. Health Policy.

[B51] Gabutti G, Penna C, Rossi M, Salmaso S, Rota MC, Bella A, Crovari P (2001). The seroepidemiology of varicella in Italy. Epidemiol Infect.

[B52] Katsafadou A, Kallergi K, Ferentinos G, Goulioti T, Foustoukou M, Papaevangelou V (2009). Presumptive varicella vaccination is warranted in Greek adolescents lacking a history of disease or household exposure. Eur J Pediatr.

[B53] Khoshnood B, Debruyne M, Lancon F, Emery C, Fagnani F, Durand I, Floret D (2006). Seroprevalence of varicella in the French population. Pediatr Infect Dis J.

[B54] Garnett GP, Cox MJ, Bundy DA, Didier JM, St Catharine J (1993). The age of infection with varicella-zoster virus in St Lucia, West Indies. Epidemiol Infect.

[B55] Brisson M, Edmunds WJ, Law B, Gay NJ, Walld R, Brownell M, Roos L, De Serres G (2001). Epidemiology of varicella zoster virus infection in Canada and the United Kingdom. Epidemiol Infect.

[B56] Kudesia G, Partridge S, Farrington CP, Soltanpoor N (2002). Changes in age-related seroprevalence of antibody to varicella zoster virus: impact on vaccine strategy. J Clin Pathol.

[B57] Deguen S, Chau NP, Flahault A (1998). Epidemiology of chickenpox in France (1991–1995). J Epidemiol Community Health.

[B58] Yawn B, Saddier P, Wollan P, St Sauver J, Kurland M, Sy L (2007). A population-based study of the incidence and complication rates of herpes zoster before zoster vaccine introduction. Mayo Clin Proc.

[B59] Menson E, Heath PT, Lyall H, Ramsay M, Sinka K, Miles J, Fleming T, Tong W, Breuer J, Nyman AG (2008). 2006–7 chickenpox season: 13 childhood deaths in England, Scotland and Wales compared with averages of 4–8.7 per year in the United Kingdom: more evidence for routine introduction of the varicella vaccine?. Arch Dis Child.

[B60] Grote V, von Kries R, Springer W, Hammersen G, Kreth HW, Liese J (2008). Varicella-related deaths in children and adolescents – Germany 2003–2004. Acta Paediatr.

[B61] Meyer PA, Seward JF, Jumaan AO, Wharton M (2000). Varicella mortality: trends before vaccine licensure in the United States, 1970–1994. J Infect Dis.

[B62] Miller E, Marshall R (1993). Epidemiology, outcome and control of varicella-zoster infection. Rev Med Microbiol.

[B63] Mohsen AH, McKendrick M (2003). Varicella pneumonia in adults. Eur Respir J.

[B64] Sauerbrei A, Wutzler P (2000). The congenital varicella syndrome. J Perinatol.

[B65] Sauerbrei A, Wutzler P (2001). Neonatal varicella. J Perinatol.

[B66] Sauerbrei A, Wutzler P (2005). Varicella-zoster virus infections during pregnancy: epidemiology, clinical symptoms, diagnosis, prevention and therapy. Curr Pediat Rev.

[B67] Thomas SL, Hall AJ (2004). What does epidemiology tell us about risk factors for herpes zoster?. Lancet Infect Dis.

[B68] Schmader K, Gnann JW, Watson P (2008). The epidemiological, clinical, and pathological rationale for the herpes zoster vaccine. J Infect Dis.

[B69] Grote V, von Kries R, Rosenfeld E, Belohradsky BH, Liese J (2007). Immunocompetent children account for the majority of complications in childhood herpes zoster. J Infect Dis.

[B70] Scott FT, Johnson RW, Leedham-Green M, Davies E, Edmunds WJ, Breuer J (2006). The burden of herpes zoster: a prospective population based study. Vaccine.

[B71] Chidiac C, Bruxelle J, Daures JP, Hoang-Xuan T, Morel P, Leplege A, El Hasnaoui A, de Labareyre C (2001). Characteristics of patients with herpes zoster on presentation to practitioners in France. Clin Infect Dis.

[B72] Davies L, Cossins L, Bowsher D, Drummond M (1994). The cost of treatment for post-herpetic neuralgia in the UK. Pharmacoeconomics.

[B73] Varicella vaccination overview in European countries. http://www.euvac.net/graphics/euvac/vaccination/var.html.

[B74] Sengupta N, Booy R, Schmitt HJ, Peltola H, Van-Damme P, Schumacher RF, Campins M, Rodrigo C, Heikkinen T, Seward J, Jumaan A, Finn A, Olcen P, Thiry N, Weil-Olivier C, Breuer J (2008). Varicella vaccination in Europe: are we ready for a universal childhood programme?. Eur J Pediatr.

[B75] Wutzler P, Schmitt H-J, Bisanz H (2008). Soziale Pädiatrie und Jugendmedizin. Kinderärztliche Praxis.

[B76] Robert Koch Institut (2007). Empfehlungen der Ständigen Impfkommission (STIKO) am Robert Koch-Institut/Stand: Juli 2007. Epidemiol Bull.

[B77] Siedler A, Arndt U (2008). Varicella sentinel in Germany shows success of vaccination. 26th Annual Meeting of the European Society for Paediatric Infectious Diseases (ESPID); 13–17 May; Graz, Austria.

[B78] Banz K, Wagenpfeil S, Neiss A, Goertz A, Staginnus U, Vollmar J, Wutzler P (2003). The cost-effectiveness of routine childhood varicella vaccination in Germany. Vaccine.

[B79] Knuf M, Neiss A, Wutzler P (2006). Impact of universal varicella vaccination in Germany: an epidemiological and economic analysis. Klin Padiatr.

[B80] Rentier B, Gershon AA, the members of EuroVar the European Working Group on Varicella (EuroVar) (2004). A consensus paper: varicella vaccination of healthy children – a challenge for Europe. Pediatr Infect Dis J.

[B81] Ramet J, Weil-Olivier C, Sedlak W (2005). Is Europe ready to embrace a policy of universal varicella vaccination?. Int J Clin Pract.

[B82] Knuf M, Habermehl P, Zepp F, Mannhardt W, Kuttnig M, Muttonen P, Prieler A, Maurer H, Bisanz H, Tornieporth N, Descamps D, Willems P (2006). Immunogenicity and safety of two doses of tetravalent measles-mumps-rubella-varicella vaccine in healthy children. Pediatr Infect Dis J.

[B83] Kuter BJ, Brown ML, Hartzel J, Williams WR, EvesiKaren A, Black S, Shinefield H, Reisinger KS, Marchant CD, Sullivan BJ, Thear M, Klopfer S, Xu J, Gress JO, Schodel F (2006). Safety and immunogenicity of a combination measles, mumps, rubella and varicella vaccine (ProQuad). Hum Vaccin.

[B84] Reynolds MA, Chaves SS, Harpaz R, AS L, Seward JF (2008). The impact of the varicella vaccination program on herpes zoster epidemiology in the United States: a review. J Infect Dis.

[B85] Nguyen HQ, Jumaan AO, Seward JF (2005). Decline in mortality due to varicella after implementation of varicella vaccination in the United States. N Engl J Med.

[B86] Banz K, Wagenpfeil S, Neiss A, Hammerschmidt T, Wutzler P (2004). The burden of varicella in Germany: potential risks and economic impact. Eur J Health Econom.

[B87] McCauley MM, Stokley S, Stevenson J, Fishbein DB (2008). Adolescent vaccination: coverage achieved by ages 13–15 years, and vaccinations received as recommended during ages 11–12 years, National Health Interview Survey 1997–2003. J Adolesc Health.

[B88] Wutzler P, Neiss A, Banz K, Goertz A, Bisanz H (2002). Can varicella be eliminated by vaccination? Potential clinical and economic effects of universal childhood varicella immunisation in Germany. Med Microbiol Immunol (Berl).

[B89] Seward JF, Watson BM, Peterson CL, Mascola L, Pelosi JW, Zhang JX, Maupin TJ, Goldman GS, Tabony LJ, Brodovicz KG, Jumaan AO, Wharton M (2002). Varicella disease after introduction of varicella vaccine in the United States, 1995–2000. JAMA.

[B90] Zhou F, Harpaz R, Jumaan AO, Winston CA, Shefer A (2005). Impact of varicella vaccination on health care utilization. JAMA.

[B91] Centers for Disease Control and Prevention (2008). Update: recommendations from the Advisory Committee on Immunization Practices (ACIP) regarding administration of combination MMRV vaccine. MMWR.

[B92] Department of Health and Human Services Centers for Disease Control and Prevention Advisory Committee on Immunization Practices Summary report, 27–28 February 2008. http://www.cdc.gov/vaccines/recs/ACIP/downloads/min-feb08.pdf.

[B93] FDA: information pertaining to labeling revision for ProQuad. http://www.fda.gov/BiologicsBloodVaccines/Vaccines/ApprovedProducts/ucm123798.htm.

[B94] Reisinger KS, Brown ML, Xu J, Sullivan BJ, Marshall GS, Nauert B, Matson DO, Silas PE, Schodel F, Gress JO, Kuter BJ (2006). A combination measles, mumps, rubella, and varicella vaccine (ProQuad) given to 4- to 6-year-old healthy children vaccinated previously with M-M-RII and Varivax. Pediatrics.

[B95] Halperin S, Guiseppe F, Scheifele D, Gerard P, Guiseppe S, Cuccia M, Willems P (2007). Immunogenicity and safety of measles-mumps-rubella-varicella vaccine given as a second dose in children up to six years of age. 25th International Congress of Paediatrics (ICP); 25–30 August; Athens, Greece.

[B96] European Medicines Agency: ProQuad summary of product characteristics, 11 September 2008. http://www.emea.europa.eu/humandocs/PDFs/EPAR/proquad/062206en6.pdf.

[B97] Bernaola Iturbe E, Gimenez Sanchez F, Baca Cots M, de Juan Martin F, Diez Domingo J, Garces Sanchez M, Gomez-Campdera A, Martinon-Torres F, Picazo JJ, Pineda Solas V (2007). Vaccination schedule of the Spanish Association of Pediatrics: recommendations 2007. An Pediatr (Barc).

[B98] Sosa LE, Hadler JL (2008). Epidemiology of varicella in Connecticut, 2001–2005. J Infect Dis.

[B99] Seward J, Marin M, Vazquez M (2008). Varicella vaccine effectiveness in the US vaccination program: a review. J Infect Dis.

[B100] Chaves SS, Gargiullo P, Zhang JX, Civen R, Guris D, Mascola L, Seward JF (2007). Loss of vaccine-induced immunity to varicella over time. N Engl J Med.

[B101] Kuter B, Matthews H, Shinefield H, Black S, Dennehy P, Watson B, Reisinger K, Kim LL, Lupinacci L, Hartzel J, Chan I (2004). Ten year follow-up of healthy children who received one or two injections of varicella vaccine. Pediatr Infect Dis J.

[B102] Nader S, Bergen R, Sharp M, Arvin AM (1995). Age-related differences in cell-mediated immunity to varicella-zoster virus among children and adults immunized with live attenuated varicella vaccine. J Infect Dis.

[B103] Watson B, Boardman C, Laufer D, Piercy S, Tustin N, Olaleye D, Cnaan A, Starr SE (1995). Humoral and cell-mediated immune responses in healthy children after one or two doses of varicella vaccine. Clin Infect Dis.

[B104] MMR vaccination overview in European countries. http://www.euvac.net/graphics/euvac/vaccination/mmr.html.

[B105] World Health Organization (2004). Measles vaccines. Wkly Epidemiol Rec.

[B106] Zepp F, Manegold-Randel D, Helm K, Gildberg P, Knuf M, de la Bourdonnaye G, Willems P (2008). Antibody persistence and varicella breakthrough case assessment three years after administration of measles-mumps-rubella-varicella (MMRV) vaccine in children aged 11–23 months. 26th Annual Meeting of the European Society of Paediatric Infectious Diseases (ESPID); 13–17 May; Graz, Austria.

[B107] Peltola H, Heinonen OP, Valle M, Paunio M, Virtanen M, Karanko V, Cantell K (1994). The elimination of indigenous measles, mumps, and rubella from Finland by a 12-year, two-dose vaccination program. N Engl J Med.

[B108] Vesikari T, Baer G, Willems P (2007). Immunogenicity and safety of a second dose of measles-mumps-rubella-varicella (MMRV) vaccine in healthy children aged 5–6 years. Pediatr Infect Dis J.

[B109] Jumaan AO, Yu O, Jackson LA, Bohlke K, Galil K, Seward JF (2005). Incidence of herpes zoster, before and after varicella-vaccination-associated decreases in the incidence of varicella, 1992–2002. J Infect Dis.

[B110] Brisson M, Gay NJ, Edmunds WJ, Andrews NJ (2002). Exposure to varicella boosts immunity to herpes-zoster: implications for mass vaccination against chickenpox. Vaccine.

[B111] Thomas SL, Wheeler JG, Hall AJ (2002). Contacts with varicella or with children and protection against herpes zoster in adults: a case-control study. Lancet.

[B112] Insinga RP, Itzler RF, Pellissier JM, Saddier P, Nikas AA (2005). The incidence of herpes zoster in a United States administrative database. J Gen Intern Med.

[B113] Hambleton S, Steinberg SP, LaRussa PS, Shapiro ED, Gershon AA (2008). Risk of herpes zoster in adults immmunized with varicella vaccine. J Infect Dis.

[B114] Hardy I, Gershon AA, Steinberg SP, LaRussa P (1991). The incidence of zoster after immunization with live attenuated varicella vaccine. A study in children with leukemia. Varicella Vaccine Collaborative Study Group. N Engl J Med.

[B115] Gershon AA, Chen J, Gershon MD (2008). A model of lytic, latent, and reactivating varicella-zoster virus infections in isolated enteric neurons. J Infect Dis.

[B116] Son M, Shapiro E, LaRussa P, Neu N, Michalik D, Meglin M, Bitar W, Jurgrau A, Flynn P, Gershon A (2008). Vaccination of children with perinatal HIV infection protects against varicella and zoster. Pediatric Academic Societies' Annual Meeting; 2–6 May; Honolulu, Hawaii.

[B117] Vaccination coverage for measles containing vaccines. http://data.euro.who.int/cisid/.

[B118] Hammerschmidt T, Bisanz H, Wutzler P (2007). Universal mass vaccination against varicella in Germany using an MMRV combination vaccine with a two-dose schedule: an economic analysis. Vaccine.

[B119] Bonanni P, Boccalini S, Bechini A, Banz K (2008). Economic evaluation of varicella vaccination in Italian children and adolescents according to different intervention strategies: the burden of uncomplicated hospitalised cases. Vaccine.

[B120] Zhou F, IR O-S, Guris D, Shefer A, Lieu T, Seward JF (2008). An economic analysis of the universal varicella vaccination program in the United States. J Infect Dis.

