# Supplementary material for: Varicella vaccination in Europe – taking the practical approach
Source: BMC Med. 2009 May 28;7:26. doi: 10.1186/1741-7015-7-26 (PMC2697173; doi:10.1186/1741-7015-7-26)
Supplement: Additional file 1 — Tables S1–S3. Table S1 – Summary of varicella-related mortality data from European countries. Table S2 – Seroprevalence of varicella among children in Europe. Table S3 – Recommendations for varicella and measles-mumps-rubella (MMR) vaccination in European countries. [file 1741-7015-7-26-S1.doc]

**Table 1.** Summary of varicella-related mortality data from European countries.

| **Country** | **Reference** | **Study sample** | **Population** | **Study period** | **Number of deaths from varicella** | **Mortality rate** |
| --- | --- | --- | --- | --- | --- | --- |
| **France** | Boelle & Hanslik, 2002 [17] | National surveillance data | Non-immune population | 1990–1999 | 19 deaths/year | 7 deaths per 1,000,000 cases (1–4 years); 104 deaths per 1,000,000 cases (25–34 years); 5345 deaths per 1,000,000 cases (>65 years) |
|  | Bonmarin *et al*., 2005 [18] | CépiDC national surveillance data | N=428 death certificates mentioning varicella or chickenpox | 1979–2000 | 18 deaths/year | NA |
|  | Mallet *et al*., 2004 [19] | Hospital discharge data from 1 paediatric centre covering area of about 400,000 inhabitants | N=309 hospitalised children (75% <2 years of age) | 1987–2002 | 2/309 (0.6%) children died during the study period | NA |
| **Germany** | Liese *et al*., 2008 [20] | ESPED active surveillance covering all paediatric hospitals and departments in Germany | N=918 hospitalised children <17 years of age | 2003–2004 | 10/918 (1.1%) children hospitalised with varicella died | 0.4 deaths per 1,000,000 children (<17 years) |
| **Greece** | Theodoridou *et al*., 2006 [21] | Discharge data from one paediatric hospital | N=498 previously healthy children <14 years of age | 1998–2002 | 1/498 (0.2%) children hospitalised with varicella died | NA |
| **Ireland and  the UK** | Cameron *et al*., 2007 [22] | Active surveillance data from the UK and Ireland | N=112 children <16 years of age hospitalised with varicella complications | 2002–2003 | 6/112 (5.4%) children hospitalised with varicella died | 0.04 per 100,000 population/year (<16 years) |
| **Italy** | Marchetto *et al*., 2007 [23] | Hospital discharge data from 3 Italian paediatric hospitals | N=349 hospitalised children <18 years of age | 2002–2006 | 1/349 (0.3%) children died during the study period | NA |
| **The Netherlands** | de Melker *et al*., 2006 [24] | Hospital admission data | Deaths from varicella reported to Netherlands’ Statistics (ICD-10) | 1996–2002 | 2 deaths per year; 50% aged <5 years | NA |
|  | Boot *et al*., 2008 [25] | Hospital admission data  Sentinel surveillance network | N=36 children hospitalised with varicella | 2006–2007 | 1/36 (2.8%) children hospitalised with varicella died | NA |
| **Slovenia** | Socan & Blasko, 2007 [26] | National surveillance data | N=9120–15538 varicella cases/year (all ages) | 1996–2005 | No deaths reported | NA |
| **Spain** | Gil *et al*., 2002 [27] | National surveillance data | N=3632 varicella-related hospital discharges (all ages) | 1995–1998 | 6/3632 (0.2%) deaths reported; average of 1.5 deaths per year; 100% immunocompetent | 1.6 deaths per 1000 varicella hospitalisations (all ages) |
|  | Gil *et al*., 2004 [28] | National surveillance data | N=5746 hospitalisations for varicella (all ages) | 1995–2000 | 48/5746 (0.8%) patients hospitalised with varicella died | 3.5 deaths per 100,000 cases |
|  | Gil *et al*., 2004 [29] | National surveillance data | N=3083 hospitalisations for varicella (all ages) | 1999–2000 | 31/3083 (1.0%) patients hospitalised with varicella died | Overall case fatality rate 1% and 3.6%, respectively |
| **Switzerland** | Bonhoeffer *et al*., 2005 [30] | Survey mailed to all 38 paediatric units in Switzerland | N=335 hospitalised patients with varicella (0–16 years of age) | 2000–2003 | 3/335 (0.9%) children hospitalised with varicella died; 66% immunocompetent | 1 death per 100,000 VZV infections in Switzerland (children 0–16 years) |
| **United Kingdom** | Rawson *et al*., 2001 [31] | Office for National Statistics | N=119 death certificates mentioning varicella or chickenpox | 1995–1997 | Average 25 deaths per year; adults accounted for 81% of deaths and 19% of consultations | 9.22 deaths per 100,000 consultations |

NA, not available; VZV, varicella zoster virus

No epidemiological data were identified via PubMed from the following European Union countries: Austria, Belgium, Bulgaria, Cyprus, Czech Republic, Denmark, Estonia, Finland, Hungary, Latvia, Lithuania, Luxembourg, Malta, Poland, Portugal, Romania and Slovak Republic.

**Table 2.** Seroprevalence of varicella among children in Europe.

| **Country** | **Reference** | **Population** | **Study period** | **Seroprevalence rate (%)** |
| --- | --- | --- | --- | --- |
| Italy | Gabutti *et al.,* 2001 [51] | 5–9 years | Sep. 1996 – Oct. 1997 | 61.8 |
| Greece | Katsafadou *et al.,* 2009 [52] | 6 years | Not stated | 63.6 |
| Belgium | Thiry *et al.,* 2002 [48] | 5 years | Oct. 1999 – Apr. 2000 | 80.2 |
| France | Khoshnood *et al.,* 2006 [53] | 7–8 years | Nov. 2003 – Jan. 2004 | 89.0 |
| The Netherlands | de Melker *et al.,* 2006 [24] | 5 years | Oct. 1995 – Dec. 1996 | 93.0 |

**Table 3.** Recommendations for varicella [6, 73-75] and measles-mumps-rubella (MMR) [104] vaccination in European countries.

|  | **Varicella vaccination recommendations** | | **MMR vaccination recommendations** | | | |
| --- | --- | --- | --- | --- | --- | --- |
| **Country** | **URV** | **Current vaccination recommendations** | **URV  (schedule type)** | **Current vaccination recommendations** | | |
|  |  |  |  | **Dose 1** | **Dose 2** | **Catch-up** |
| **Austria** | 🗶 | Seronegative girls/women of childbearing age; seronegative health care workers (especially in paediatric institutions); high-risk children (e.g. children with forthcoming transplantation or chemotherapy or immunosuppression, before immunosuppression); seronegative family members of high-risk children; seronegative day-care personnel and teachers | ✓ (accelerated) | 11–23 months | 28 days after dose 1 at 12–24 months | 7–16 years for unvaccinated children |
| **Belgium** | 🗶 | High-risk patients | ✓ (longer) | 12 or 13 months | 10–13 years | 5–7 years for MMR dose 1 and at 14–16 years for MMR dose 2 |
| **Bulgaria** | 🗶 | None | ✓ (longer) | 13 months | 11–12 years |  |
| **Croatia** | 🗶 | None | ✓ (standard) | 12–18 months | 6 years |  |
| **Cyprus** | 🗶 | Childhood immunization from 13 months in the private sector only | ✓ (standard) | 12–15 years | 4–6 years | 11–12 years |
| **Czech Republic** | 🗶 | None | ✓ (accelerated) | 15 months | 21–25 months |  |
| **Denmark** | 🗶 | None | ✓ (standard) | 15 months | 4 years | 12 years |
| **Estonia** | 🗶 | None | ✓ (longer) | 12 months | 13 years |  |
| **Finland** | 🗶 | On an individual named patient basis | ✓ (standard) | 14–18 months | 6 years |  |
| **France** | 🗶 | High-risk groups with no varicella history: post-exposure vaccination in adolescents; women of child-bearing age or after first pregnancy; healthcare workers; seronegative close contacts of immunosuppressed; in children 6 months prior to receiving solid grafts | ✓ (accelerated) | 12 months  1–9 months (children in daycare) | 13–24 months  12–15 months (children in daycare) | Infants 25 months to children 15 years of age |
| **Germany** | ✓ | Administered according to a 2-dose schedule to all children at 11–14 months and 15–23 months of age as MMRV or monovalent varicella vaccines | ✓ (accelerated) | 11–14 months | 15–23 months |  |
| **Greece** | ✓ | Administered according to a 2-dose schedule to all children at 12–18 months and 4–6 years of age | ✓ (standard) | 15 months | 4–6 years |  |
| **Hungary** | 🗶 | On an individual named patient basis | ✓ (longer) | 15 months | 11 years |  |
| **Iceland** | 🗶 | None | ✓ (longer) | 18 months | 12 years |  |
| **Ireland** | 🗶 | None | ✓ (standard) | 12–15 months | 4–5 years | 11–12 years |
| **Italy** | 🗶 | Priority to all susceptible adults and adolescents, and then possibly all children living in regions able to reach high coverage rates (>80%) in the short-term (according to regional programmes)  Sicily: universal childhood vaccination in second year of life and catch-up in 12-year-olds with no history of varicella | ✓ (standard) | 12–24 months | 5–6 years | 12 years |
| **Latvia** | 🗶 | None | ✓ (standard) | 15 months | 7 years | 11–12 years |
| **Lithuania** | 🗶 | None | ✓ (standard) | 15–16 months | 6–7 years | 12 years |
| **Luxembourg** | 🗶 | None | ✓ (standard) | 15–18 months | 5–6 years |  |
| **Malta** | 🗶 | None, but considering introducing recommendations for childhood immunisation to be administered with first dose of MMR vaccine | ✓ (longer) | 15 months | 8–9 years |  |
| **The Netherlands** | 🗶 | None | ✓ (longer) | 14 months | 9 years |  |
| **Norway** | 🗶 | None | ✓ (longer) | 15 months | 12–13 years |  |
| **Poland** | 🗶 | Recommended for all susceptible individuals | ✓ (longer) | 13–14 months | 10 years | 11–12 years for all previously unvaccinated females |
| **Portugal** | 🗶 | None | ✓ (standard) | 15 months | 5–6 years |  |
| **Romania** | 🗶 | None | ✓ (standard) | 12–15 months | 6–7 years |  |
| **Slovak Republic** | 🗶 | None | ✓ (longer) | 14 months | 10 years |  |
| **Slovenia** | 🗶 | None | ✓ (standard) | 12–24 months | 5–6 years |  |
| **Spain** | ✓ (Madrid) | No official recommendation, but a 2-dose schedule at 12–18 months and 3–4 years, and catch-up vaccination of susceptible adolescents at 11–12 years recommended by the Spanish Association of Pediatrics  Madrid Autonomous Region: universal childhood vaccination in infants aged 15 months | ✓ (standard) | 12–15 months | 3–6 years |  |
| **Sweden** | 🗶 | High-risk groups; seronegative healthy children >12 years of age and adults who have not had varicella | ✓ (standard) | 18 months | 6–8 years for children born in 2002 or later or at 12 years for children born before 2002 |  |
| **Switzerland** | 🗶 | Seronegative adolescents aged 11–15 years  Catch-up for persons with no history of varicella | ✓ (accelerated) | 12 months | 15–24 months |  |
| **Turkey** | 🗶 | None | ✓ (standard) | 12 months | 6 years | Rubella at 13 years for all unvaccinated females |
| **United Kingdom** | 🗶 | Non-immune healthcare workers; healthy close contacts of immunosuppressed patients; on an individual named patient basis | ✓ (standard) | 13 months | 3 years 4 months to 5 years |  |

MMR, measles-mumps-rubella; MMRV, measles-mumps-rubella-varicella vaccine; URV, universal routine vaccination
