# Supplementary material for: Varicella vaccination in Europe – taking the practical approach
Source: BMC Med. 2009 May 28;7:26. doi: 10.1186/1741-7015-7-26 (PMC2697173; doi:10.1186/1741-7015-7-26)
Supplement: Additional file 2 — Supplementary appendix. Summary of varicella epidemiology in European countries. [file 1741-7015-7-26-S2.doc]

# Supplementary appendix. Summary of varicella epidemiology in European countries.

| **Country** | **Notifications** | | **Hospitalisations** | | **Complications** | **Study population** | **Reference** |
| --- | --- | --- | --- | --- | --- | --- | --- |
|  | **Total (all ages)** | **Age adjusted** | **Total (all ages)** | **Age adjusted** |  |  |  |
| **France** | 1255 cases /100,000 population/year | 4973 cases /100,000 population/year (aged <1 year); 12,124 cases /100,000 population/year (aged 1–4 years) |  | 23.0/100,000 population (aged <15 years); 28.0/100,000 population (aged 1–4 years); 4.9/100,000 population (aged 25–34 years); 0.8/100,000 population (aged ≥65 years) |  | Individuals with varicella (all ages) identified in nationwide databases and sentinel surveillance systems between 1990 and 1999 | Boelle & Hanslik, 2002 [19] |
|  | 22 cases/100,000 population/year |  | 5.8/100,000 population/year | 15,294/21,179 (72.2%) of hospitalisations (total between 1997 and 2004) were in children <10 years of age | 7058/21,179 (33.3%) of hospitalised varicella cases (reported between 1997 and 2004) were accompanied by complications (cutaneous and pulmonary complications were the most common) | Individuals with varicella (all ages) identified in a sentinel surveillance network between 1990 and 2004 (incidence), individuals (all ages) hospitalised with varicella identified by discharge codes between 1997 and 2002, N=21,179 varicella cases (hospitalisations and complications) | Bonmarin *et al*., 2005 [20] |
|  |  |  |  | 28/100,000 population (aged <16 years); 149/100,000 population (aged <1 year); 2/100,000 population  (aged 5–15 years) | 93/162 (57.4%) of hospitalised varicella cases were accompanied by complications (44/93 [47.3%] experienced a skin infection) | Children (aged <16 years) hospitalised with varicella in 11 hospitals with paediatric units in Northern France, 2003–2004, N=162 varicella admissions | Dubos *et al*., 2007 [34] |
|  |  |  |  | 6 (0.6%) children aged <13 years visiting a GP were hospitalised  Mean duration of hospital stay was 4.1 days | 90/1152 (7.8%) cases visiting a GP had complications (most common was bacterial superinfection, 50/1152 patients [4.3%]) | Individuals with varicella visiting a GP, N=1152 cases of varicella (94% <13 years of age) | Emery *et al*., 2006 [35] |
|  |  |  |  | Median duration of hospital stay was 5 days  38/1575 (2.4%) of hospitalisations required intensive care | 1200/1575 (76.2%) of hospitalisations were due to a complication (bacterial superinfection was the most common, 792/1575 of patients [50.3%]) | Paediatric patients hospitalised with varicella, between March 2003 and July 2005, N=1575 cases of varicella | Grimprel *et al*., 2007 [36] |
|  |  |  | Median duration of hospital stay was 4.5 days |  | 75% of complications in hospitalised infants <2 years of age | Hospitalised patients with varicella; retrospective study in a hospital with a catchment area of 400,000 inhabitants, 1987–2002 | Mallet *et al*., 2004 [21] |
| **Germany** |  |  |  | 14.1/100,000 children (aged ≤16 years); 710/918 (77.3%) of patients were previously healthy  Median duration of hospital stay was 5 days | Complications occurred in 730/913 (80.0%) of hospitalisations, most commonly neurologic (25.4%), skin infections (23.2%), and gastrointestinal (15.0%)  93/918 (10.1%) patients hospitalised for varicella had permanent or possible permanent long-term sequelae | Children (aged ≤16 years) admitted to a paediatric hospital for varicella recruited from active surveillance, N=918 cases | Liese *et al*. 2008 [22] |
|  | 760,000 cases/year | Highest incidence in children aged 5–6 years | Median duration of hospital stay was 5.3 days |  | Complications occurred in 76 patients (population weighted varicella rate of 5.7%). Bacterial superinfection, otitis media, pneumonia and bronchitis were most common in children aged <12 years | 1334 unvaccinated varicella cases from paediatric and general practices (90% <12 years of age) | Wagenpfeil *et al*., 2004 [37] |
|  |  |  |  |  | 8.5 complications/100,000 population aged <16 years  Most frequent complication among hospitalised patients: neurological (73/119, 61.3%), infectious (46/119, 38.6%)  8/119 (6.7%) hospitalised patients had long-term sequelae | Children (aged ≤16 years) admitted to a paediatric hospital for varicella recruited from surveillance, N=119 cases of varicella | Ziebold *et al*., 2001 [38] |
| **Greece** |  |  |  | 761/48,781 (1.6%) of school children with a positive varicella history had been hospitalised for varicella-related complications |  | Survey of 70,226 parents of 1^st^ and 6^th^ grade school children. | Katsafadou *et al*. 2008 [39] |
|  |  |  |  | 15.3/100,000 population (aged <15 years) | Most frequent reasons for varicella hospitalisation were skin infection (106/498, 21.3%), respiratory complication (87/498, 17.5%) and neurological complication (82/498, 16.5%)  2/498 (0.4%) patients experienced long-term sequelae due to infectious complications of varicella (deep vein thrombosis and necrotising fasciitis) | Hospitalised paediatric patients aged <15 years with varicella, N=498 | Theodoridou *et al*., 2006 [23] |
| **Ireland** |  | 2.32 cases/100,000 population (aged 0–4 years);  0.54 cases/100,000 population (aged 5–9 years);  0.12/100,000 population (aged 9–15 years) |  | Mean duration of hospital stay (aged <16 years) was 7 days  29/112 (25.9%) of children hospitalised were admitted to intensive care (rate, 0.21/100,000 population/year) | A concurrent or secondary bacterial infection was reported in 52/112 (46.4%) of patients hospitalised with varicella | Paediatric patients (aged <16 years) hospitalised with varicella in the UK and Ireland identified via paediatrician reporting to surveillance systems, 2002–2003, N=112 cases of varicella | Cameron *et al*., 2007 [24] |
| **Italy** |  | 5345 cases/100,000 children aged <15 years |  |  |  | Paediatric sentinel survey of 468 paediatricians (covering 371,670 children aged <15 years) | Ciofi Degli Atti *et al*., 2002 [40] |
|  |  |  |  | 13/349 (3.7%) hospitalised children had serious underlying diseases | 261/349 (74.8%) of hospitalisations were due to a complication, including neurological disorders (100/261, 38.3%) | Children (aged <18 years) hospitalised with varicella identified by discharge codes between 2002 and 1996, N=349 | Marchetto *et al*., 2007 [25] |
| **The Netherlands** | 254 cases /100,000 population/year |  | 1.3/100,000 population (varicella as main diagnosis)  2.3/100,000 population (varicella main and side diagnosis)  Mean duration of hospital stay was 6.5 days |  |  | Individuals with varicella (all ages) identified in GP surveillance systems between 2000 and 2002, N=8359 (incidence); individuals hospitalised with varicella identified by discharge codes between 1994 and 2001 (hospitalisations) | de Melker *et al*., 2006 [26] |
|  | 300 cases/100,000 population/year |  | 2.0/100,000 population (varicella as main diagnosis, data from 2006)  2.9/100,000 population (varicella main and side diagnosis, data from 2006)  Mean duration of hospital stay was 7.2 days | Incidence highest in infants aged <1 year,  58.2 per 100,000 population (varicella as the main diagnosis) | 37 complications were reported via paediatric surveillance unit, 2006–2007 (further details available for 36 patients)  32/36 (88.9%) patients hospitalised experienced ≥1 complication, most commonly bacterial or viral superinfection (19/36, 52.8%) | Individuals with varicella (all ages) identified in GP surveillance systems between 2000 and 2006, N=8359 (incidence); individuals hospitalised with varicella identified by discharge codes (hospitalisations and complications) | Boot *et al*., 2008 [27] |
| **Poland** | 371 cases/100,000 population (2006) | Urban area: 3874/100,000 population (aged 5–9 years); 2779/100,000 (aged 1–4 years)  Rural area: 3036/100,000 population (aged 5–9 years); 1580/100,000 (aged 1–4 years) | 948 cases hospitalised in 2006 |  |  | Notified cases of varicella (all ages) in nationwide surveillance | National Institute of Hygiene/Chief Sanitary Inspectorate, 2006 [41] |
| **Romania** | 316 cases/100,000 population (2004) | 147,822/300,477 (49.2%) of patients hospitalised with varicella between 2000 and 2004 were in children aged <10 years |  |  | 163/371 (43.9%) of patients hospitalised with varicella were admitted with one or more complication  Common complications in children were: bacterial skin infections (58/198, 29.3%), interstitial pneumonia (35/198, 17.7%)  Common complications in adults were: thrombocytopaenia (23/173, 13.3%), bacterial skin infections (15/173, 8.7%) | Individuals with varicella (all ages) identified in nationwide databases and surveillance systems between 1986 and 2004, N=300,477 varicella cases  Individuals hospitalised with varicella between 2003 and 2004, N=371 (198 children, 127 adults) hospitalisations for varicella | Arama *et al*., 2005 [42] |
| **Slovenia** | 456–777 cases/100,000 population/year |  | 5.8 /1000 cases |  | Rate of varicella-related complications was 2.1/1,000,000 population for meningitis/meningo-encephalitis complications and 0.8/1,000,000 population for pneumonia complications | Individuals with varicella identified via surveillance between 1996 and 2005 | Socan & Blasko, 2007 [28] |
| **Spain** |  | 95.9% of cases in children <10 years; 6.9% in infants <1 year |  |  | 101/683 (14.8%) of children visiting the primary care physician had complications, most commonly skin superinfection (61/683, 8.9%), respiratory tract infection (31/683, 4.5%) and eye infection (15/683, 2.2%) | Children with varicella aged <15 years under the care of primary care physicians identified between March and June 2000, N=683 cases of varicella | Díez-Domingo *et al*., 2003 [43] |
|  |  |  | 2.8/100,000 population  Mean duration of hospital stay was 6.8 days | 2121/3632 (58.4%) of varicella-related discharges in children <10 years of age, 1194/3632 (32.9%) in adults 21–50 years old |  | Individuals with varicella (all ages) identified in national hospital surveillance systems between 1995 and 1998, N=3632 cases of varicella | Gil *et al*., 2002 [29] |
|  |  |  | 4.1/100,000 population  Varicella resulted in 11,141 days of hospitalisation/year |  | 275/1542 (17.8%) of hospitalised patients had ≥1 varicella-specific complication | Individuals with varicella (all ages) identified in national hospital surveillance systems between 1999 and 2000, N=1572 cases of primary varicella | Gil *et al*., 2004 [31] |
|  | 742.5–1239.6/100,000 person-years |  |  |  | 364/9856 (3.7%) of varicella cases reported to GP or primary care physicians resulted in complications, most commonly cutaneous complications in 68.8% patients with complications | Individuals with varicella reported by GP or primary care physicians (Madrid Sentinel General Practitioner Network) between 1997 and 2004, N=9856 cases of varicella | Pérez-Farinos *et al*., 2007 [11] |
|  |  |  |  | 12.9 /100,000 children aged <15 years; 80% of children hospitalised were <5 years; mean duration of hospital stay was 6.5 days | 42/71 (59.2%) patients hospitalised with varicella experienced complications, had bacterial superinfection | Individuals (<15 years) hospitalised with varicella complications identified by discharge codes between 1993 and 2002 (covering 54,999 children <15 years) | Pérez-Yarza *et al*., 2003 [44] |
| **Switzerland** | 77,084 cases/year throughout Switzerland |  |  | 13.0/10,000 cases; mean duration of hospital stay was 8 days | 11/335 (3.3%) of hospitalised cases required intensive care, due to haemorrhagic varicella (n=1), pneumonia (n=1), septic arthritis and toxic shock syndrome (n=1), congenital varicella with encephalitis (n=1), meningo-encephalitis (n=2), pneumococcal sepsis (n=1), and varicella pneumonitis (n=1), generalised seizures (n=2) and aspiration pneumonia (n=1) | Hospitalised paediatric patients (0–16 years)with varicella identified via national surveillance, N=335 cases | Bonhoeffer *et al*., 2005 [32] |
| **United Kingdom** | 1291/100,000 person–years |  | 4.5 /100,000 person-years  Mean duration of hospital stay was 3 days | 1527/2189 (70%) of hospitalisations in children <15 years |  | Individuals with varicella consulting a GP in England and Wales between 1991 and 2000, N=670,868 cases (incidence), individuals hospitalised with varicella identified via sentinel surveillance between 1995 and 1996, N=2190 patients hospitalised | Brisson *et al*., 2003 [45] |

No epidemiological data were identified via PubMed from the following European Union countries: Austria, Belgium, Bulgaria, Cyprus, Czech Republic, Denmark, Estonia, Finland, Hungary, Latvia, Lithuania, Luxembourg, Malta, Portugal and Slovak Republic.
